# Supplementary material for: Learning curve and surgical outcome of robotic assisted colorectal surgery with ERAS program
Source: Sci Rep. 2022 Nov 29;12:20566. doi: 10.1038/s41598-022-24665-w (PMC9709162; doi:10.1038/s41598-022-24665-w)
Supplement: Supplementary file 2 — Supplementary Information 2. [file 41598_2022_24665_MOESM2_ESM.zip › RAL Raw data-Ñ[▒K/080/Patho_ñw╝╨░O▒Kñσ.pdf]

臺中榮民總醫院 臺中市臺灣大道四段1650號 病理部活體組織切片 臨床醫學實驗及病理診斷中心 檢驗(查)報告

[redacted]

Pathologic diagnosis:
1. Rectum, robotic low anterior resection --- Adenocarcinoma, moderately differentiated. Tumor invaded to submucosa. The bilateral cut ends and perirectal lymph nodes (0/47) are free of tumor.
2. IMA root, dissection --- Unremarkable fibrovasculoadipose tissue.
3. Rectum, cut end, distal, resection --- Unremarkable.

Prognostic and predictive factor:
1. Tumor size: 1.2x0.6 cm.
2. Depth of invasion:Submucosa.
3. Histologic grade: G2: Moderately differentiated.
4. Angiolymphatic invasion: Not identified.
5. Perineural invasion: Not identified.
6. Tumor deposits (discontinuous extramural extension): Not identified.
7. Macroscopic tumor perforation: Not identified.
8. Surgical Margins:
a) Proximal Margin: Uninvolved by invasive carcinoma, distance of tumor from margin: 14.2 cm.
b) Distal Margin: Uninvolved by invasive carcinoma, distance of tumor from margin: 2.2 cm.
c) Radial or Mesenteric Margin: Uninvolved by invasive carcinoma, distance of tumor from margin: 3 cm.
9. Lymph node status: Uninvolved (0/47).
10. Extranodal involvement: Not applicable.
11. Tumor regression grading S/P CCRT: No known presurgical therapy.
12. Type of polyp in which invasive carcinoma arose: Not identified.
13. Additional pathologic findings: Hyperplastic polyps.
14. Pathological TNM Stage: pT1N0 (According to the eighth edition, American Joint Committee on Cancer Staging Guidelines for Tumors).
15. TNM descriptors: Not applicable.

Gross description:
The specimen consists of 1) a segment of colon, 17.6 cm in length and 3.8 cm in proximal cut end circumference, 6.4 cm in distal circumference, and perirectal soft tissue. A tan and soft polypoid lesion, 1.2x0.6 cm, is noted, 3.2 cm to the distal cut end. On cross section, no definite invasion is seen grossly with more than 4 cm to the radial margin. Numerous small polypoid lesions are also seen over the colon mucosa, up to 0.2x0.2 cm. The both cut ends are free of lesion grossly. 2) IMA root, a piece of tan yellow soft tissue, 2.5x1.2x0.3 cm. 3) distal cut end, a piece of tan mucosa-covered soft tissue, 1.7x1.7x0.6 cm. Representative part for section: A-N) specimen 1 [A: proximal cut end, B-C: polypoid lesion, D: radial margin, E: flat mucosa close to polypoid lesion, F-H: colon mucosa with smaller polyps, L-N: lymph nodes] J) specimen 2 K) specimen 3.

Note:
This case has been peer reviewed by two doctors.
#T-59600_2 #M-81403_2 2 1105 B0000F
#T-C4560_2 #M-09183_2 0 1105 000000

已電子簽章

敬告：本單僅供醫師參考使用，不具任何申請效力！！
請病患妥為保管，若不慎遺失需自負個人資料外洩責任！
